# Evaluation of dysphagia in different phenotypes of early and idiopathic Parkinsonism

**DOI:** 10.1186/s41983-018-0031-1

**Published:** 2018-10-24

**Authors:** Al-Amir Bassiouny Mohamed, Gharib Fawi Mohamed, Hassan Mohamed Elnady, Mohamed Abdelmoneim Sayed, Ahmed Mamdouh Imam, Megahed Mohamed Hassan, Sherihan Rezk Ahmed

**Affiliations:** 1 Department of Neurology and Psychological Medicine, Sohag University, Sohag Governorate, Egypt; 2 Phoniatric Unit, Faculty of medicine, Department of Otolaryngology, Sohag University, Sohag Governorate, Egypt

**Keywords:** Parkinsonism, Dysphagia, Swallowing disturbance questionnaire, Fiberoptic endoscopic evaluation of swallowing

## Abstract

**Background:**

Parkinsonism (PD) is a common neurodegenerative disorders into which dysphagia occurs mainly in the late stage and to a lesser extent in an early stage. Diagnosis of dysphagia particularly in early idiopathic Parkinson’s disease (IPD) is important as dysphagia affects the quality of life of patients and most of the patients are unaware of this important symptom.

**Method:**

Fifty-four patients were enrolled in this study presented with early IPD attending to the outpatient clinic of Sohag University Hospital. All PD patients were assessed by using Unified Parkinson’s Disease Rating Scale (UPDRS) and modified Hoehn and Yahr scale. IPD patients were classified into tremor dominant (TD) and postural instability/gait disorder (PIGD) phenotypes. Swallowing disturbance questionnaire (SDQ) and fiberoptic endoscopic evaluation of swallowing (FEES) were used to evaluate dysphagia.

**Results:**

Thirty-five percent of patients experienced dysphagia when the patients were questioned, and this percent rises to 40% on using FEES. The results of SDQ were significantly correlated to the results of more accurate FEES. The percentage of dysphagia was higher in patients with PIGD than TD phenotype. Dysphagia was significantly associated with the mean of the Mini-Mental State Examination (MMSE), UPDRS, and modified Hoehn and Yahr scale.

**Conclusions:**

Dysphagia is a prevalent symptom in early IPD and significantly correlated with Parkinsonism phenotype, UPDRS, and modified Hoehn and Yahr scale.

**Electronic supplementary material:**

The online version of this article (10.1186/s41983-018-0031-1) contains supplementary material, which is available to authorized users.

## Background

Parkinson’s disease (PD) is a common neurodegenerative disorder with prevalence 140 per 100,000 [1, 2].

Most of the patients with PD develop dysphagia in the late stage of the disease in spite of early pathological changes of brainstem structures [3–6].

Dysphagia in IPD may occur in up to 100% of the patients in advanced disease stages [7, 8] which impairs the quality of life, interferes with medication intake, and leads to malnutrition and aspiration pneumonia, which is the major cause of death in PD [9].

A recent meta-analysis showed that the prevalence of oropharyngeal dysphagia is 35% on using subjective tools like questionnaires, and this percent increases to 82% by using the more objective measures like FEES [7].

Fiberoptic endoscopic evaluation of swallowing (FEES) is considered one of the gold standard tests for the diagnosis of dysphagia, which can be replaced by a simple questionnaire like SDQ when FEES is not available [10].

Previous studies reported that patients with postural instability/gait disorder (PIGD) have greater problems of swallowing than tremor dominant phenotype (TD) which indicate a positive relationship between dysphagia and range of motor skills, including falling, postural instability, and gait dysfunction [11, 12].

To the best of our knowledge, most of the studies underestimate the frequency and severity of dysphasia in radiologically documented dysphagia, and the simple, sensitive, and specific bedside tests available for diagnosis of dysphagia in IPD are lacking [13–16].

This cross-sectional study was done to detect dysphagia in early and idiopathic parkinsonism by using SDQ and the more accurate FEES and to evaluate the frequency of dysphagia in different phenotypes of IPD as the early detection of dysphagia reduces bad impact on the quality of life of patients.

## Methods

### Patients

The present observational study was conducted on 54 consecutive PD patients (38 men, 16 women, mean age 62.30 ± 5.6) whom regularly followed up at the outpatient clinic of the Sohag University Hospital from June 2013 to February 2016. They continued levodopa drugs during the time of the study, and all patients were in the early stages of PD and were evaluated in their best on state.

The work was approved by the Medical Research, Ethical Committee of Sohag Faculty of Medicine on September 7, 2014, with reference number (75). All participants were informed in detail about the investigation, and informed written consent was obtained.

All patients fulfilled the UK Parkinson’s Disease Brain Bank criteria for PD [17]. Unified Parkinson’s Disease Rating Scale (UPDRS) [18] and the modified Hoehn and Yahr scale [19] were used during on state to evaluate the severity and stage of IPD, respectively. Mini-Mental State Examination (MMSE) [20] was used to evaluate the cognitive status of the patients with IPD.

The following inclusion criteria were applied: Patients fulfilled the UK Brain Bank diagnostic criteria of PD [17], with modified Hoehn and Yahr (modified H&Y) stage between 1 and 3 to target early Parkinson’s disease. Patients with MMS examination > 24 (as cognitive impairment affects the reliability of SDQ), hearing problems, and pharyngoesophageal local conditions were excluded from this study.

To identify PD phenotypes, patients were classified as tremor dominant (TD), postural instability/gait disorder (PIGD), or indeterminate according to the previously described formula that uses items from the UPDRS where the ratio of the mean UPDRS tremor scores (8 items) to the mean UPDRS PIGD scores (5 items) was used to define TD patients (ratio ≥ 1.5), PIGD patients (ratio ≤ 1), and indeterminate patients (ratios > 1.0 and < 1.5) [21, 22].

### Study design and ethics

This cross-sectional study was carried out on patients with idiopathic PD to evaluate the dysphagia in early stage of PD and to assess dysphagia in different phenotypes of IPD.

### Methods

All patients were evaluated by a neurologist and sent to a speech therapist to evaluate dysphagia by FEES and complete a 15-item questionnaire on swallowing disturbances (Additional file 1) [3]. If the score of swallowing disturbance questionnaire (SDQ) is more than or equal to 11, this indicates dysphagia [3].

Also, speech therapist calculated the drooling score by using Drooling Severity and Frequency Scale (DSFS) Appendix 1 [23].

### Objective evaluation of dysphagia by fiberoptic endoscopy

Evaluation of dysphagia in IPD patients during on state was made by fiberoptic endoscopic evaluation of swallowing (FEES). This FEES was carried out by using fiberoptic nasopharyngolaryngoscope (Model 20045020, Storz, Germany). Two examiners made the examination: a phoniatrician who did the endoscopic examination and a nurse who feed the patient by a spoon. Three types of food were offered: (1) solid component by using bread soaked with yogurt, (2) semisolid using yogurt, and (3) liquid using 100 ml of water colored with green food coloring material (1 g.) The endoscope passed through the nose, choana, and nasopharynx and stopped just below the level of the palate in order to visualize the oropharynx, hypopharynx, and the larynx on a monitor. At the moment of swallowing, the pharyngeal wall collapsed and obliterated the pharyngeal cavity. So, the swallow event could not be seen. However, the examiner can comment on events just before and after the swallow. These are initiation delay/residue, penetration, and aspiration. Detailed comments should focus on (1) initiation delay or residue: in the mouth, vallecullae, pyriform sinuses, or in the pharyngeal walls; (2) penetration: food or liquid entering the airway entrance (above or at the vocal fold level); and (3) aspiration: food or liquid entering the airway to the level of trachea (below the level of vocal folds) [24].

### Statistics

The statistical analysis was performed using the Statistical Package for the Social Sciences (SPSS 20.0, SPSS Inc., Chicago, IL, USA) for windows. Descriptive statistics were done to investigate the general characteristics of the patients. Chi-square was used for correlation between qualitative data while Pearson’s correlation was used for continuous data. Student’s *t* test was performed to explore the effect of abnormal swallowing on characteristics of patients with PD. Logistic regression analysis was performed to find out independent predictors of dysphagia. All the results were considered significant when *P* < 0.05.

## Results

The mean age of the 54 participants in the study (38 men and 16 women) was 62.3 (age range 51–75 years). We selected our participants in the early stage of the disease with mean disease duration 4.7 ± 2.2. The mean of UPDRS part II score and UPDRS part III score was 15.5 ± 8.9 and 37.5 ± 16.3, respectively, while modified Hoehn and Yahr rating was 2.1 ± 0.6.

**Fig. 1:**
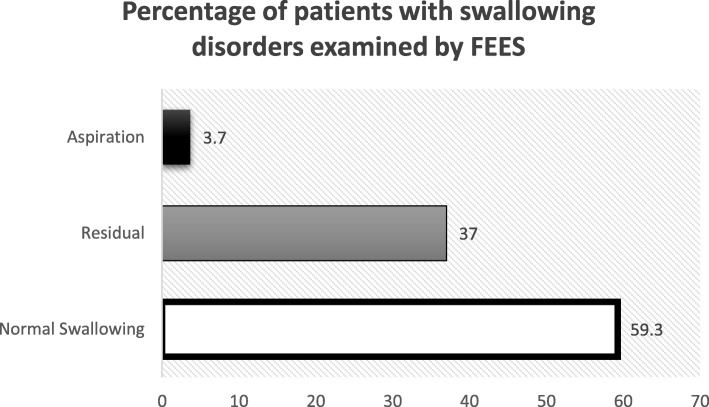
Percentage of patients with swallowing disorders examined by FEES

When the PD patients were questioned, the percentage of dysphagia was 35.1% particularly the oral phase which nearly doubles the percentage of the pharyngeal phase and this percentage increased to 40.7% when using the more accurate and objective method (FEES) (Table 1 and Fig. 1).

**Table 1:** Baseline characteristics of patients with PD

	Mean	Standard deviation (SD)
Age (mean ± SD)	62.30	5.642
Diagnosis		
Disease duration (mean ± SD)	4.7	2.2
Disease severity		
Mini-Mental State Examination (MMSE) (30 = normal)	26.8	1.8
Unified Parkinson’s Disease Rating Scale (UPDRS)		
UPDRS part II score (68 = severe)	15.5	8.9
UPDRS part III score (108 = severe)	37.5	16.3
Modified Hoehn and Yahr rating (5 = severe)	2.1	0.6
	Number of patients	Percentage
Sex		
Male	38	70.4
Female	16	29.6
Parkinsonism phenotype		
Tremor dominant	46	85.2
Postural Instability and gait instability	8	14.8
Swallowing disturbance questionnaire		
Normal	35	64.8
Dysphagia	19	35.2
Oral phase	13	24
Pharyngeal phase	6	11.1
FEES		
Normal swallowing	32	59.3
Residual	20	37.0
Aspiration	2	3.7
Salivation scale		
Normal salivation	17	31.5
Sialorrhea	37	68.5

The mean age of patients with normal swallowing and dysphagia was 63.5 ± 5.9 versus 60.5 ± 4.8, respectively, with a *P* value of 0.05.

Most of the patient with dysphagia have a longer disease duration (5.3 ± 2.1) than those with normal swallowing (4.3 ± 2.1) with a *P* value equal to 0.1.

The percentage of dysphagia in PIGD and TD type was 75 and 34%, respectively, with a *P* value equal to 0.02.

SDQ was significantly correlated with the objective FEES (*P* value < 0.001) which means that the simple SDQ can be a screening tool replacing the less available FEES Table 2.

**Table 2:** Comparison of the baseline characteristics between subjects with and without dysphagia based on FEES

	Normal swallowing *N* = 32	Dysphagia *N* = 22	*P* value
Age (mean ± SD)	63.50 ± 5.9	60.55 ± 4.8	0.058
Sex			0.753
Male	22 (57.9%)	16 (42.1%)	
Female	10 (62.5%)	6 (37.5%)	
Disease duration (mean ± SD)	4.38 ± 2.1	5.36 ± 2.1	0.106
Disease severity			
Parkinsonism phenotype			0.022
Tremor dominant	30 (65.2%)	16 (34.8%)	
Postural instability and gait instability	2 (25.0%)	6 (75.0%)	
SDQ			< 0.001
Normal	28 (87.5%)	7 (31.8%)	
Dysphagia	4 (12.5%)	15 (68.2%)	
Salivation			0.5
Normal salivation	9 (28.1%	8 (36.4%)	
Sialorrhea	23 (71.9%)	14 (63.6%)	
MMSE (mean ± SD)	27.56 ± 2.0	25.8 ± 1.0	0.001
UPDRS			
UPDRS part II score (mean ± SD) (68 = severe)	11.25 ± 3.0	21.91 ± 10.9	0.000
UPDRS part III score (mean ± SD) (108 = severe)	35.81 ± 6.2	40.00 ± 24.5	0.359
UPDRS total (mean ± SD)	55.19 ± 10.4	78.09 ± 33.8	0.001
Modified Hoehn and Yahr scale (mean ± SD)	1.813 ± .5	2.727 ± .4	0.000

*MMSE* Mini-Mental State Examination, *UPDRS* Unified Parkinson’s Disease Rating Scale, *SDQ* swallowing disturbance questionnaire

The mean MMS examination in dysphagia group is significantly lower (but still within normal range) than the normal group (25.8 ± 1.0 versus 27.56 ± 2.0 with a *P* value = 0.001).

Dysphagia was significantly correlated with UPDRS part II score, UPDRS part III score, and modified Hoehn and Yahr scale Table 2.

**Fig. 2:**
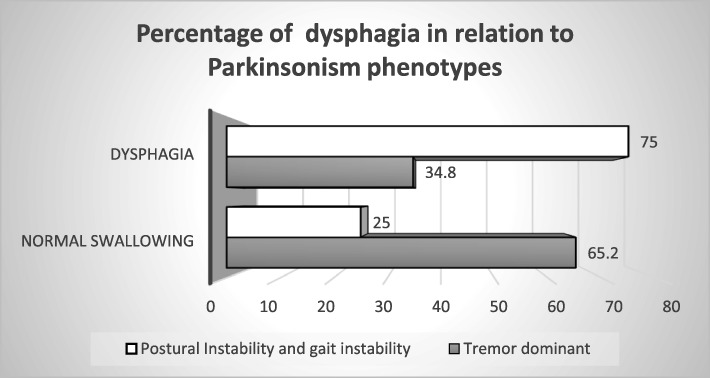
Percentage of dysphagia in relation to parkinsonism phenotypes

Parkinsonism phenotype and activity of daily living (ADL) were independent predictors of dysphagia Table 3 and Fig. 2.

**Table 3:** Predictors of dysphagia in PD

	*B*	S.E.	Wald	df	Sig.	Exp(*B*)	95% C.I. for EXP(*B*)	
							Lower	Upper
Parkinsonism phenotype	1.947	0.913	4.543	1	0.033	7.007	1.170	41.973
Total UPDRS	− 0.042	0.037	1.271	1	0.260	.959	0.891	1.032
II_ADL	0.330	.144	5.232	1	0.022	1.391	1.048	1.846
Sialorrhea	0.426	0.664	.411	1	0.521	1.530	.417	5.619
Constant	− 3.546	1.675	4.480	1	0.034	0.029		

*UPDRS* Unified Parkinson’s Disease Rating Scale, *ADL* activity of daily living

Most of our series have TD phenotype, but the percentage of dysphagia is significantly higher in PIGD phenotype Table 4.

**Table 4:** Dysphagia in relation to different phenotypes of parkinsonism

	Tremor dominant (TD) *N* = 46	Postural instability and gait instability (PIGD) *N* = 8	*P* value
SDQ			0.342
Normal	31 (67.4%)	4 (50.0%)	
Dysphagia	15 (32.6%)	4 (50.0%)	
Salivation			0.041
Normal	12 (26.1%)	5 (62.5%)	
Sialorrhea	34 (73.9%)	3 (37.5%)	
FEES			0.033
Normal	30 (65.2%)	2 (25.0%)	
Dysphagia	16 (34.8%)	6 (75.0%)	

*SDQ* swallowing disturbance questionnaire, *FEES* fiberoptic endoscopic evaluation of swallowing

## Discussion

Normal swallowing depends on smooth coordination of the oral and pharyngeal phase. An impaired oral phase may impair the airway during the pharyngeal phase [25, 26].

The prolonged oral transit time which may be a factor that predispose to dysphagia in PD, a finding reported from a radiological study [27].

Aspiration and pneumonia are common complications of dysphagia and may occur at any stage of PD but more with advanced stage [28].

More than 35% of patients experience dysphagia on SDQ, and this percentage increases to about 40% when using FEES which is similar to the results of previous studies which reported that the occurrence of dysphagia is between 20 and 40% in patients with IPD [16] in contrast to previous work of Manor and colleagues which stated a higher percentage of dysphagia (63.2%) [3]; this difference may be related to the selection of patient as most of our participants are outpatients, and their H&Y scale was less than 3 which is classified as an early stage of IPD. Also, the mean duration of illness in our PD patients was 4.7 which means a relatively short duration of PD.

Oral phase of dysphagia is more commonly affected than pharyngeal phase (24% versus 11.1% respectively) like other studies which confirmed the affection of oral phase more than pharyngeal phase of swallowing in PD [29].

Like previous studies, the present study revealed that dysphagia is significantly correlated with UPDRS and modified Hoehn and Yahr scale [30, 31] in contrast to the others [32, 33] which showed that the disease stage (UPDRS total and Hoehn and Yahr rating) was not correlated to swallowing difficulties which may be explained by the relatively early stage of PD in our patients (H&Y scale< 3), the shorter duration of illness (4.7 ± 2.2) and the usage of different scores for evaluation of dysphagia.

The present work emphasized the greater percentage of dysphagia in PIGD group than tremor dominant group (75% versus 34.8% respectively). This finding was in line with the previous studies which reported that dysphagia may be associated with more “axial” motor disturbance, increased postural instability, and more falls [11, 12].

The mechanism of dysphagia in PD may be related to extrapyramidal and autonomic system disorders into which degeneration of the dorsal nucleus of the vagus and esophageal myenteric plexus is responsible for dysphagia [34], and this neurodegeneration in the autonomic nervous system occurring not only in the late stage but also in the early stages of PD [35]; this finding may be confirmed by the fact that dysphagia is partially improved by dopaminergic therapy [12, 35, 36].

Our results showed a strong correlation between PD severity on UPDRS and dysphagia, so the severity could be a predictor of dysphagia in PD patients like many previous studies with apparently larger sample sizes which documented the significant association between the severity of PD and dysphagia [15, 25, 26]. On the other hand, Ali and Wallace did not observe any correlation between the clinical severity of PD and dysphagia, which may be explained by a smaller sample size of their participants and most of their patients had advanced PD (H&Y stage more than III) [37].

The major limitation of this study was the relatively small sample size and lack of follow-up of patients with dysphagia especially the asymptomatic group and the effect of different dopaminergic drugs on dysphagia.

In spite of these limitations, the present study has shown that dysphagia is a common symptom even in an early stage of PD. The simple and available SDQ may be a useful screening tool for detection of dysphagia in PD and the patients with abnormal SDQ better to be referred to a speech therapist.

## Conclusions

In conclusion, the present work is one of few studies that exhibited the association between dysphagia and early PD and showed that dysphagia symptoms is more prevalent in PIGD phenotype. Dysphagia could be easily detected by SDQ and confirmed by FEES as the early detection of dysphagia decreases the deleterious impact on quality of life of PD patients.

### Additional files

Additional file 1: Swallowing disturbance questionnaire. (DOCX 15 kb)

## Appendix 1

Drooling Severity and Frequency Scale (DSFS) considering the sum of the scores for severity (1, dry: never drools; 2, mild: only lips wet; 3, moderate: lips and chin wet; 4, severe: clothing soiled; 5, profuse: clothing, hands, and tray moist wet) and frequency (1, never drools; 2, occasional drooling—not every day; 3, frequent drooling—every day; 4, constant drooling) [23].

## Abbreviations

FEES: Fiberoptic endoscopic evaluation of swallowing
IPD: Idiopathic Parkinson’s disease
MMSE: Mini-Mental State Examination
Modified H&Y: Modified Hoehn and Yahr
PD: Parkinson’s disease
PIGD: Postural instability/gait disorder
SDQ: Swallowing disturbance questionnaire
SPSS: Statistical Package for the Social Sciences
TD: Tremor dominant
UPDRS: Unified Parkinson’s Disease Rating Scale
