# Supplementary material for: Evaluation of dysphagia in different phenotypes of early and idiopathic Parkinsonism
Source: Egypt J Neurol Psychiatr Neurosurg. 2018 Oct 24;54(1):28. doi: 10.1186/s41983-018-0031-1 (PMC6208737; doi:10.1186/s41983-018-0031-1)
Supplement: Supplementary file 1 — Swallowing disturbance questionnaire. (DOCX 15 kb) [file 41983_2018_31_MOESM1_ESM.docx]

**Additional file 1: Swallowing Disturbance Questionnaire** ^[1]^

| **Questions** | **0** | **1** | **2** | **3** |
| --- | --- | --- | --- | --- |
|  | Never | Seldom (**once** a month or less) | Frequently (**1–7**  times a week) | Very frequently (> **7** times a week) |
| 1. Do you experience difficulty chewing solid food like an apple, cookie or a cracker? |  |  |  |  |
| 2. Are there any food residues in your mouth, cheeks,  under your tongue or stuck to your palate after swallowing? |  |  |  |  |
| 3. Does food or liquid come out of your nose when you eat or drink? |  |  |  |  |
| 4. Does chewed up food dribble from your mouth? |  |  |  |  |
| 5. Do you feel you have too much saliva in your mouth; do you drool or have difficulty swallowing your saliva? |  |  |  |  |
| 6. Do you swallow chewed up food several times before it goes down your throat? |  |  |  |  |
| 7. Do you experience difficulty in swallowing solid food (i.e., do apples or crackers get stuck in your throat)? |  |  |  |  |
| 8. Do you experience difficulty in swallowing pureed food? |  |  |  |  |
| 9. While eating, do you feel as if a lump of food is stuck in your throat? |  |  |  |  |
| 10. Do you cough while swallowing liquids? |  |  |  |  |
| 11. Do you cough while swallowing solid foods? |  |  |  |  |
| 12. Immediately after eating or drinking, do you experience a change in your voice, such as hoarseness or reduced? |  |  |  |  |
| 13. Other than during meals, do you experience coughing or  difficulty breathing as a result of saliva entering your  windpipe? |  |  |  |  |
| 14. Do you experience difficulty in breathing during meals? |  |  |  |  |
| 15. Have you suffered from a respiratory infection (pneumonia,bronchitis) during the past year? | Yes | No |  |  |

Five questions related to the oral phase of swallowing and 10 questions related to the pharyngeal phase. Fourteen questions were rated by a four-point (0–3) scale (0 for no disability and 3 for severe disability) and one was a “yes/no” question (yes was scored 2.5 and no was scored 0.5)

1. Manor Y, Giladi N, Cohen A, Fliss DM, Cohen JT. Validation of a swallowing disturbance questionnaire for detecting dysphagia in patients with Parkinson's disease. Movement Disorders. 2007;22(13):1917-21.
